# Equivalent Electromechanical Model for Quartz Tuning Fork Used in Atomic Force Microscopy

**DOI:** 10.3390/s23083923

**Published:** 2023-04-12

**Authors:** Rui Lin, Jianqiang Qian, Yingzi Li, Peng Cheng, Cheng Wang, Lei Li, Xiaodong Gao, Wendong Sun

**Affiliations:** 1 School of Physics, Beihang University, Beijing 100191, China; 2 Fujian Engineering and Research Center of Green and Environment-Friendly Functional Footwear Materials, College of Chemical Engineering and Materials Science, Quanzhou Normal University, Quanzhou 362000, China

**Keywords:** atomic force microscope, quartz tuning fork, higher eigenmode, electromechanical model

## Abstract

Quartz tuning forks (QTFs) are self-sensing and possess a high quality factor, allowing them to be used as probes for atomic force microscopes (AFMs) for which they offer nano-scale resolution of sample images. Since recent work has revealed that utilizing higher-order modes of QTFs can offer better resolution of AFM images and more information on samples, it is necessary to understand the relationship between the vibration characteristics of the first two symmetric eigenmodes of quartz-based probes. In this paper, a model that combines the mechanical and electrical characteristics of the first two symmetric eigenmodes of a QTF is presented. Firstly, the relationships between the resonant frequency, amplitude, and quality factor between the first two symmetric eigenmodes are theoretically derived. Then, a finite element analysis is conducted to estimate the dynamic behaviors of the analyzed QTF. Finally, experimental tests are executed to verify the validity of the proposed model. The results indicate that the proposed model can accurately describe the dynamic properties of a QTF in the first two symmetric eigenmodes either under electrical or mechanical excitation, which will provide a reference for the description of the relationship between the electrical and mechanical responses of the QTF probe in the first two symmetric eigenmodes as well as the optimization of higher modal responses of the QTF sensor.

## 1. Introduction

Cantilevers have been the most frequently used sensors since the invention of atomic force microscope (AFM) [1]. Generally, an AFM’s tip is integrated with a cantilever. When the tip approaches the surface of the sample, the tip–sample interaction causes the deflection of the cantilever, which can be detected and translated into the surface information of the sample. With the development of AFM sensing technology, a quartz tuning fork (QTF) was first used as an AFM probe in 1989 due to its self-sensing properties and high quality factor (Q-factor) value [2]. Since then, quartz-based probes have been regarded as highly promising in the fields of atomic force microscopy [3,4,5], quartz-enhanced photoacoustic spectroscopy (QEPAS) [6,7], etc.

By simultaneously exciting the first two eigenmodes of the probe, bimodal atomic force microscopy offers an optimal method with which to obtain more information, such as the mechanical or electrical properties of samples, with just one scan [8]. With the development of bimodal atomic force microscopy, many multi-frequency methods, such as band excitation [9], frequency intermodulation [10], mode synthesizing [11], and dual resonance frequency tracking [12], have been presented and implemented in the highly sensitive nano-detection of samples [13]. In this case, it is necessary to understand the vibration properties of the QTF in the first two eigenmodes, which will help improve the detection sensitivity or resolution in bimodal atomic force microscopy.

Unlike a cantilever probe, which has only one arm, a QTF has two arms that are highly symmetrical. A quartz-based probe generally consists of a QTF and tip(s) glued to one or both QTF arms. When only one tip is glued to one arm of the QTF, the symmetry of the QTF probe is nullified, which will reduce the Q-factor. In the qPlus configuration, one arm of the QTF is bonded to a block, while the other is attached to the tip [14]. This configuration is similar to that of a cantilever probe, so the vibration theory is similar to the cantilever beam theory [15]. On the contrary, when both arms of the QTF are glued with the same tips, the high symmetry of the QTF can be maintained, and the Q-factor can be improved via comparison with the qPlus configuration.

Due to a QTF’s two-arm structure and piezoelectric properties, its vibration has a greater degree of complexity, e.g., the coupling between the two arms and the relationship between the electrical and mechanical characteristics. To investigate the complex electromechanical response of the QTF probe, Oria et al. developed a finite element model of a QTF that is excited electrically [16,17]. Chen et al. introduced a QTF probe with a much slighter glass tip attached, which maintained a high Q-factor by minimizing the effect of asymmetry [4]. The effects of certain parameters on the resonant performance of a QTF probe were investigated by finite element analysis and experimental tests in [18]. Lee et al. presented a comprehensive electromechanical model for a QTF to analyze and compare the dynamic responses of an electrically driven QTF and qPlus sensor [19].

However, regarding higher eigenmodes of QTF, understanding the dynamic characteristics and detection sensitivity of the QTF at higher modes is vital. Kim et al. analyzed the first seven eigenmodes of a QTF through finite element method (FEM) simulations and experiments and proposed a modified single-beam theory [20]. Tung et al. established a theoretical model of a quartz sensor and tested its vibration properties through laser Doppler vibrometry to study the higher modes of a qPlus sensor and found that the geometry of the tips has a great influence on the vibration properties of the quartz sensor [15]. Zhang et al. investigated the sensing performance of a QTF at the second eigenmode based on FEM and experimentation [21]. Chen et al. presented a numerical analysis method for analyzing the vibration behavior of a qPlus sensor with a long tip [22].

This paper focuses on the transformation relationship between the electrical and mechanical behaviors of the first two eigenmodes of a currently used QTF. Section 2 introduces a comprehensive electromechanical model of the QTF that can describe the vibration properties of its first two eigenmodes. Section 3 presents the FEM simulation on the frequency response to the first two eigenmodes of the QTF. Then, the experimental tests and discussions are addressed in Section 4. Finally, our conclusions are presented in Section 5.

## 2. Electromechanical Model of QTF

**Figure 1:**
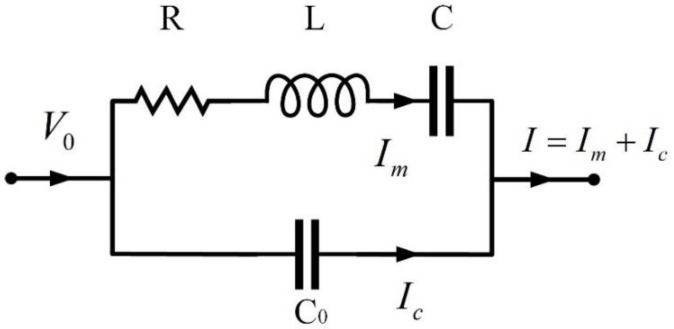
Equivalent circuit model of QTF.

A QTF has piezoelectric and inverse piezoelectric properties. As a consequence, it can be treated as an equivalent RLC oscillating circuit. It can be found that the linear motion of a QTF can be described by an RLC circuit, and recent research has indicated that the nonlinearity of a QTF sensor cannot be neglected [23]. Therefore, the equivalent circuit model of a QTF can be regarded as an RLC connected with parallel capacitance, $C_{0}$, as shown in Figure 1.

The total signal, *I*, can be expressed as follows:

(1) $$I=I_{m}+I_{c}=\left(\frac{1}{R+j\omega L-j\frac{1}{\omega C}}-\frac{1}{j\frac{1}{\omega C_{0}}}\right)V_{0}$$

where $I_{m}$ represents the current of the RLC circuit, which describes the vibration motion of the QTF, and $I_{c}$ represents the stray current induced by the parallel capacitance $C_{0}$. *j* represents an imaginary unit in the RLC circuit in the same way as *i* operates in mathematics, where *j*^2^ = *i*^2^ = −1. Based on the equivalent circuit model, the resonance curve function can be derived as follows:

(2) $$A_{e}=\left|\frac{A_{0}\omega }{Q_{1}\omega_{1}}\left(\frac{1}{1-\frac{\omega^{2}}{\omega_{1}^{2}}+j\frac{\omega }{\omega_{1}Q_{1}}}+{\bar{C}}_{0}\right)\right|$$

(3) $$A_{0}=R_{out}(V_{0}/R)$$

(4) $$\omega_{1}=1/\sqrt{LC}$$

(5) $$Q_{1}=L\omega_{1}/R$$

(6) $${\bar{C}}_{0}=C_{0}/C$$

where $A_{e}$ represents the oscillation amplitude and $R_{out}$ represents the resistance that adjusts the output signal. $\omega_{1}$ and $Q_{1}$ represent the resonant angular frequency and the Q-factor of the first symmetric eigenmode, respectively.

**Figure 2:**
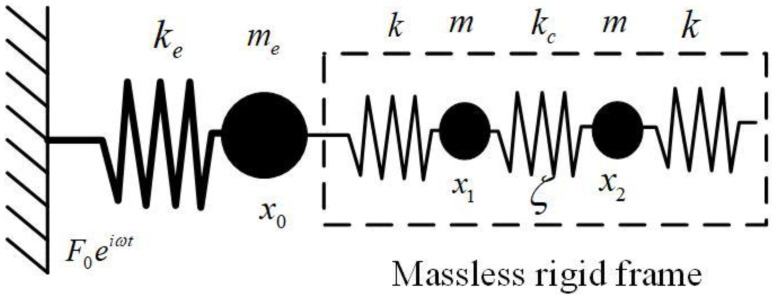
Equivalent mechanical model of QTF, where $k_{e}$ and $m_{e}$ represent the equivalent stiffness and equivalent mass of the QTF base, respectively. k and m are the equivalent stiffness and mass of each QTF arm, respectively. $k_{c}$ represents the coupled stiffness between the two arms.

As for mechanical model of the QTF, numerous models with base damping and mass have been proposed [24,25] since Naber et al. proposed a two-masses-three-springs model [26]. A mechanical model called the four-springs-three-point-masses system was also proposed [27], as shown in Figure 2. Accordingly, in this study, the applied force is expressed as $F_{0}e^{i\omega t}$, and the damping coefficient is denoted by *ζ*.

Based on the mechanical model, the motion equation of QTF can be derived as

(7) $$m{\ddot{x}}_{1}+\zeta {\dot{x}}_{1}+k\left(x_{1}-x_{0}\right)=F_{0}e^{i\omega t}$$

(8) $$m{\ddot{x}}_{2}+\zeta {\dot{x}}_{2}+k\left(x_{2}-x_{0}\right)=F_{0}e^{i\omega t}$$

Assuming that the vibration of the QTF is highly symmetric, then $x_{0}=0$, and $x_{1}=x_{2}$. Thus, the resonant frequency and Q-factor of the first two symmetric eigenmodes of QTF can be derived as

(9) $$\omega_{n}=\sqrt{k_{n}/m}$$

(10) $$Q_{n}=k_{n}/\left(\zeta \omega_{n}\right),n=1,2,$$

since both eigenmodes exhibit symmetric mode shapes.

By solving Equations (7) and (8), we obtained

(11) $$x_{1}=x_{2}=\frac{1}{k-m\omega^{2}+i\zeta \omega }F_{0}e^{i\omega t}=\frac{1}{1-\frac{\omega^{2}}{\omega_{n}^{2}}+i\frac{\omega }{\omega_{n}Q_{n}}}\frac{F_{0}e^{i\omega t}}{k_{n}}$$

**Figure 3:**
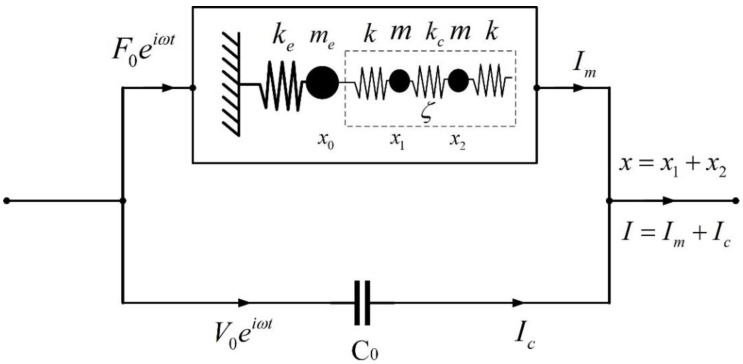
Equivalent electromechanical model for a QTF.

By combining the equivalent circuit model and the mechanical model, an equivalent electromechanical model of the QTF was obtained, as shown in Figure 3.

In this model, two coefficients, *α* and *β*, are introduced. The factor *α* is the coefficient that converts excitation voltage into the mechanical force applied to the QTF, which can be expressed as $F_{0}e^{i\omega t}=\alpha V_{0}i\omega e^{i\omega t}$. *β* is the coefficient that converts the geometrical displacements of the two arms into electrical current, which can be described as $I_{m}=\beta x$.

Considering the symmetric eigenmodes of a QTF, the displacements of the QTF base and two arms can be expressed as $x_{0}=0$ and $x_{1}=x_{2}$. Consequently, the electrical signal induced by the QTF motion is $I_{m}=2\beta x_{1}$. The total current signal can be derived as

(12) $$I=I_{m}+I_{c}=2\beta x_{1}+j\omega C_{0}V_{0}e^{i\omega t}$$

while the amplitude of output voltage signal can be derived as

(13) $$A_{n}=\left|\left[\left(\frac{1}{1-{\left(\frac{\omega }{\omega_{n}}\right)}^{2}+i\left(\frac{\omega }{\omega_{n}Q_{n}}\right)}+\frac{C_{0}k_{n}}{2\alpha \beta }\right)\frac{2\alpha \beta \omega V_{0}R_{out}}{k_{n}}\right]\right|,n=1,2$$

It can be seen from Equation (13) that when the excitation frequency is equal to the resonant frequency, i.e., $\omega =\omega_{n},(n=1,2)$, the amplitude reaches its peak value. For the mechanical peak amplitude $A_{m,n}$, it can be described as the first term of Equation (13), given that $\omega =\omega_{n},(n=1,2)$:

(14) $$A_{m,n}=\frac{2\alpha \beta \omega_{n}Q_{n}V_{0}R_{out}}{k_{n}},n=1,2.$$

The second term of Equation (13) reflects the normalized capacitance:

(15) $${\bar{C}}_{0}=\frac{C_{0}k}{2\alpha \beta }$$

Moreover, an intrinsic constant can be derived from Equation (13), which is defined as follows:

(16) $$N=\frac{\alpha \beta R_{out}}{k}=\frac{A_{n}}{2\omega_{n}Q_{n}V_{0}}$$

The proposed electromechanical model includes both mechanical and electrical parameters, which can be used to explain the relationship between the mechanical and electrical vibration characteristics of QTFs under different excitation methods. Moreover, the changes in dynamic characteristics such as the peak amplitude and Q-factor following the changes in the eigenmodes can also be explained by the model, which will be of great importance to multi-mode measurements of QTFs [28]. 

Furthermore, finite element analysis and experimental tests of the QTF were carried out to verify the validity of the proposed model.

## 3. Finite Element Analysis

**Figure 4:**
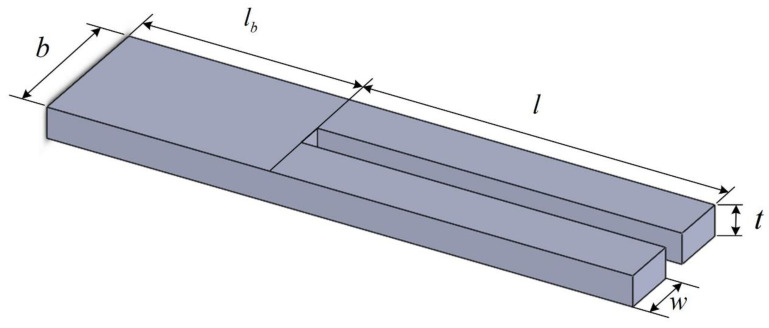
Geometry and dimensions of QTF.

The analyzed QTF’s motions and eigenfrequencies were analyzed via FEM using the SolidWorks module and the ANSYS Workbench 18.2 software package. The geometry of the QTF is depicted in Figure 4. The structure of the QTF consists of one base and two arms, whose geometric parameters are listed in Table 1. The density of the quartz is 2730 kg/m^3^. The QTF’s Young’s modulus is 79.7 GPa and its Poisson’s ratio is 0.33. 

**Figure 5:**
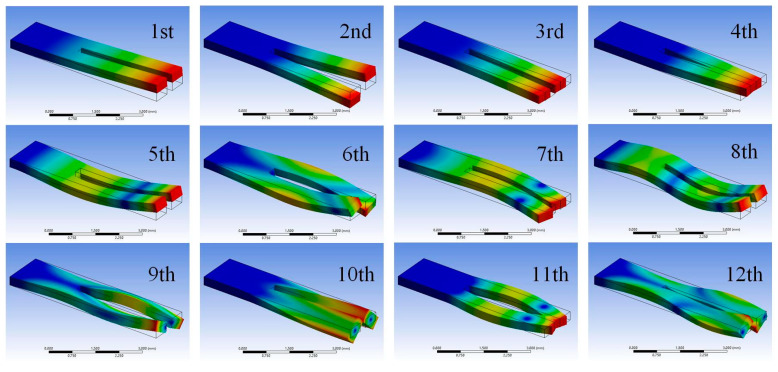
FEM simulations of the first 12 eigenmodes of QTF. The change in color from blue to red indicates the change in displacement from small to large.

Firstly, modal analysis was carried out to find the first two in-plane symmetric bending modes of the QTF, for which the bottom of the QTF’s base was fixed and its two arms were set as free ends. The first 12 eigenmodes of the QTF determined via modal analysis are shown in Figure 5, and the corresponding resonant frequencies are shown in Table 2. It was found that the fundamental in-plane symmetric bending mode is depicted as the fourth eigenmode, which has a resonant frequency of 31.380 kHz, whereas the eleventh eigenmode exhibits a second in-plane symmetric bending mode, with a resonant frequency of 183.39 kHz. According to the given geometric and physical parameters, we can obtain the mass of each arm for the QTF, i.e., *m* = 1.7 × 10^−6^ kg. From Equations (9) and (10), the equivalent stiffness can be calculated as *k*_1_ = 66 kN/m and *k*_2_ = 2257 kN/m, and the Q-factors can be calculated as *Q*_1_ = 7839 and *Q*_2_ = 11,520. Therefore, the peak amplitude ratio between the first two symmetric eigenmodes can be calculated from Equation (13), yielding *A*_1_/*A*_2_ = 11.95.

**Figure 6:**
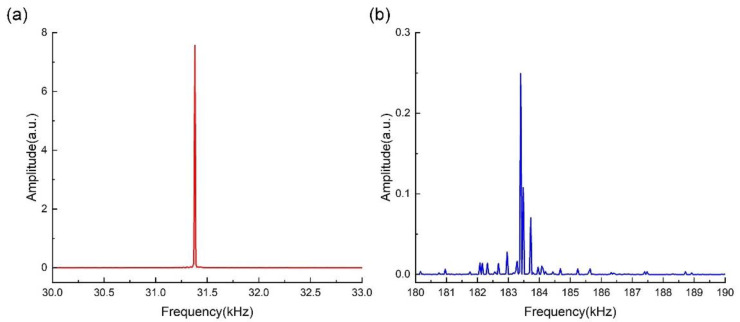
Harmonic response simulation of the (**a**) first and (**b**) second symmetric bending modes of QTF.

In addition, a harmonic response simulation was executed to analyze the amplitude–frequency responses to the first two in-plane symmetric bending modes. The simulation results are shown in Figure 6.

As shown in Figure 6, it was found that the resonant frequencies of the first two symmetric bending modes are 31.38 kHz and 183.40 kHz, respectively, which are close to the simulation results of the modal analysis. The normalized amplitudes of the first two modes are 2.84 a.u. and 0.249 a.u., respectively. Thus, we can determine that the ratio of the amplitude between the first two modes is approximately 11.41. Moreover, the Q-factors can be obtained from the resonant curve, corresponding to *Q*_1_ = 7550 and *Q*_2_ = 12,694. A comparison between the calculation and harmonic simulation results is shown in Table 3.

In Table 3, it is evident that the resonant parameters of the QTF obtained through the simulation are consistent with the calculation results. Furthermore, it is necessary to conduct experimental tests to assess the validity of the proposed model and the simulation results.

## 4. Experimental Tests

### 4.1. Setup

**Figure 7:**
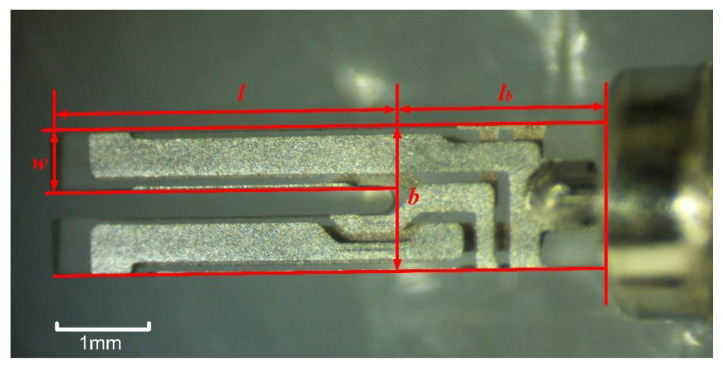
Structure of QTF used for experimental tests. The geometric parameters are listed in Table 1, including the thickness, *t*, which is not depicted in the figure.

The structure of the QTF used for the experimental tests is shown in Figure 7. The geometric parameters of the tested QTF are given in Table 1.

**Figure 8:**
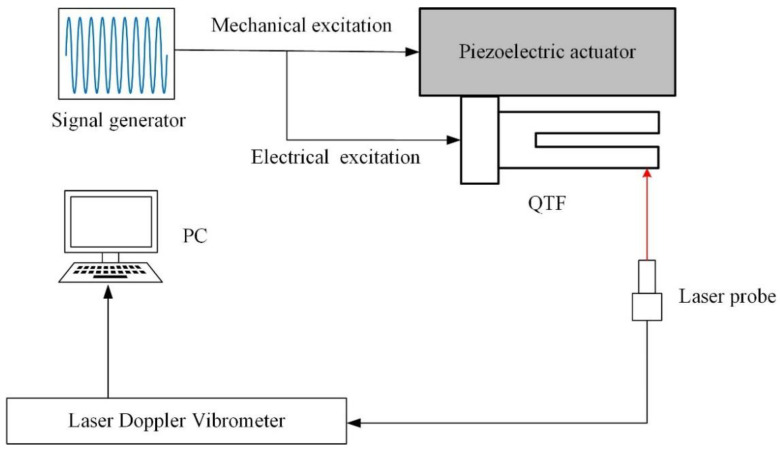
Experimental setup of QTF-testing system.

The experimental setup for measuring the dynamic responses of the QTF is depicted in Figure 8. The setup consists of a signal generator (Agilent 33250A, Agilent Technologies, Inc., Beijing, China), a piezoelectric actuator (NAC2024, Harbin Core Tomorrow Science & Technology Co., Ltd., Harbin, China), a commercial QTF (32.768 K, Shenzhen Jinghong Electronics Co., Ltd., Shenzhen, China), and a laser Doppler vibrometer (LV-S01, Yuyao Sunny Optical Intelligent Technology Co., Ltd., Yuyao, China). The QTF can be mechanically excited using the piezoelectric actuator and electrically excited by applying the excitation signal directly to the QTF itself, and its displacement is measured by the vibrometer with a displacement resolution of 0.008 nm.

### 4.2. Dynamic Response Analysis

**Figure 9:**
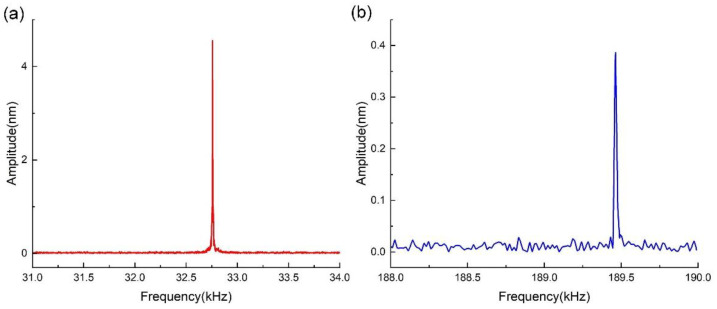
Frequency responses of the (**a**) first and (**b**) second symmetric modes of mechanically excited QTF.

Firstly, the QTF was mechanically excited using the piezoelectric actuator. The peak-to-peak value of the applied voltage was 700 mV. The dynamic responses of the QTF are shown in Figure 9. The response of the first two in-plane symmetric modes of the QTF are depicted with red and blue curves in Figure 9a,b, respectively. As shown in Figure 9, we obtained the resonant frequency and amplitude of the first symmetric bending mode, which were equal to 32.758 kHz and 4.552 nm, respectively. The resonant frequency and amplitude of the second mode were 189.463 kHz and 0.386 nm, respectively. Moreover, the Q-factors of the first and second modes were calculated as 8190 and 11,710, respectively.

**Figure 10:**
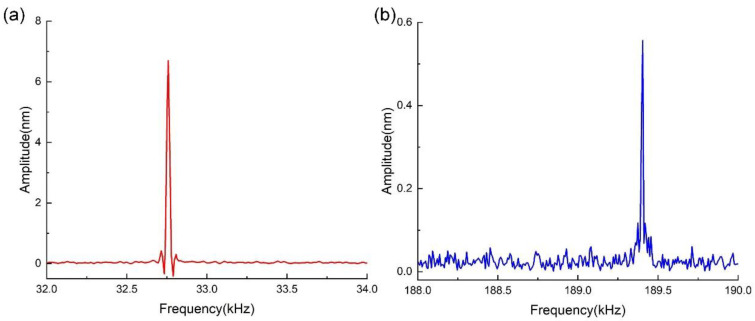
Frequency responses of the (**a**) first and (**b**) second symmetric modes of directly electrically actuated QTF.

Additionally, the QTF was actuated electrically by applying voltage with a peak-to-peak value of 700 mV, and the dynamic responses are shown in Figure 10. As shown in Figure 10, we found that when directly exciting the QTF electrically, the resonant frequency and amplitude of the first symmetric bending mode were 32.759 kHz and 6.693 nm, respectively. The resonant frequency and amplitude of the second mode were equal to 189.404 kHz and 0.556 nm, respectively. The Q-factors of the first and second modes were calculated as 8120 and 17,376, respectively.

**Figure 11:**
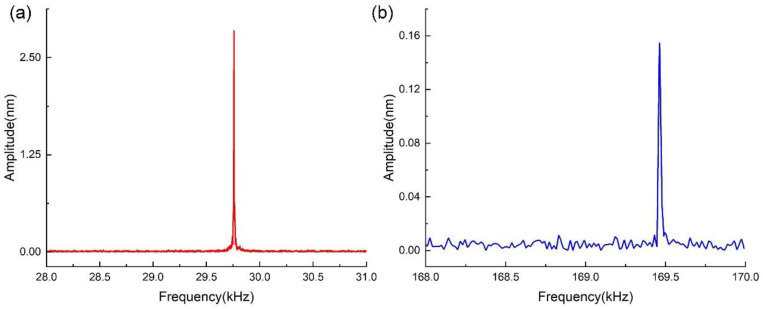
Frequency responses of the (**a**) first and (**b**) second symmetric modes of mechanically excited QTF glued with the same two tips glued on both arms.

The vibration characteristics of the QTF with the same tips glued to both arms were also tested. By applying the same voltage (700 mV) to the piezoelectric actuator to mechanically excite the QTF with tips, we obtained its frequency responses, as shown in Figure 11. The resonant frequency and amplitude of the first symmetric bending mode were equal to 29.755 kHz and 2.845 nm, respectively. The resonant frequency and amplitude of the second mode were equal to 169.485 kHz and 0.154 nm, respectively. Moreover, the Q-factors of the first and second modes were calculated as 5110 and 8527, respectively. 

### 4.3. Discussion

Firstly, we obtained the first 12 eigenmodes of the QTF via simulation through modal analysis, as shown in Figure 5. Only two of them were detected in the experimental tests, i.e., the fourth eigenmode and the eleventh eigenmode, which are considered the first two symmetric bending modes of a QTF. This is because these two modes have a relatively higher degree of symmetry than the others, which results in a much lower level of energy dissipation. It can be seen from Figure 9 and Figure 10 that the obtained resonant frequencies of the two modes induced by electrical actuation are similar to the results obtained by mechanical excitation, while the obtained amplitudes are slightly larger. One can assume that the efficiency of the electrical actuation method is higher than that of mechanical excitation since the electromechanical conversion efficiency of s piezoelectric actuator is not 100%, which may lead to energy dissipation during mechanical excitation.

We will now discuss the amplitude ratio between the first and the second symmetric bending modes. When mechanical excitation is applied, it can be found from Figure 9 that, under the condition of the same actuation magnitude, the amplitude ratio of the first mode to the second mode is approximately 11.79. Similarly, when electrical actuation is applied, it can be seen from Figure 10 that the ratio is approximately 12.04. This indicates that given the same magnitude of actuation, the amplitude ratio between the two modes is close to 12.0 independent of the actuation method.

In addition, we can find that despite the different actuation methods, the Q-factor of the second mode is higher than that of the first mode, which implies that the QTF is more sensitive at higher modes and could be more vulnerable to changes in the ambient environment. When the QTF was actuated using an electrical method, the Q-factor of the second mode was higher than when actuated by a mechanical method. This may be due to energy loss when applying mechanical excitation, which is similar to the amplitude issue mentioned above.

The resonant characteristics of the QTF obtained by calculations, simulation, and experimental tests are listed in Table 4. Test 1 refers to the experimental test wherein the QTF was excited mechanically, while Test 2 refers to the test in which it was electrically excited. Test 3 refers to the experimental test wherein the QTF with the same two tips glued on both arms was excited mechanically. By comparison, it is evident that the calculation and simulation results are consistent with the results of Test 1 and Test 2. The deviations may stem from the difference in the geometric and physical parameters. This indicates the applicability of the proposed electromechanical model. 

By comparing the results of Test 3 with the results of Test 1 and Test 2, it is evident that there is a decrease in the resonant frequencies and Q-factors of both modes. It is not difficult to understand the cause of the decrease in the resonant frequencies and amplitude according to the proposed electromechanical model of the QTF since the QTF with the same tips glued to both arms can be regarded as the QTF with a larger mass of each arm (which is indicated as *m* in Figure 3). By analyzing Equations (13) and (14), it can be derived that a larger *m* is associated with lower resonant frequencies and a smaller amplitude. Moreover, the attached tips affected the vibration symmetry of the QTF to some extent, which can explain the decrease in the Q-factor value. The increase in mass and the decrease in the Q-factor make it much more difficult to excite the second symmetric eigenmode of the QTF, which means the amplitude *A*_2_ would become much smaller, while the amplitude ratio *A*_1_/*A*_2_ would be larger.

## 5. Conclusions

In this paper, a model capable of describing both the mechanical and electrical properties of the first two eigenmodes of a QTF was proposed. By combining the mechanical motion and the electrical response of the two arms of the QTF, the proposed model can estimate the changes in the resonant frequencies, amplitude, and Q-factor from the first mode to the second mode. The applicability of the proposed model has been verified through simulations and experiments. In addition, the impact of the actuation method on the amplitude ratio between the two modes has been discussed. The results indicate that given the same actuation voltage, the amplitude ratio between the two modes is independent of the actuation method. The proposed electromechanical model provides a reference for a description of the relationship between the electrical and mechanical responses of a QTF probe in the first two symmetric eigenmodes as well as the optimization of the higher modal responses of quartz-based AFM probes.

The goal of this paper was to investigate the dynamic characteristics of the first two symmetric eigenmodes of a QTF by establishing its equivalent electromechanical model. There are still several issues to be considered, such as enhancing the responses of higher-order modes, improving the detection sensitivity of the QTF, and converting the displacement of the QTF into a detectable electric signal for atomic force microscopy imaging. Future work will focus on enhancing the higher-order modal responses of the QTF and applying them to bimodal atomic force microscopy imaging.

## Figures and Tables

**Table 1:** Geometric parameters of QTF.

Parameters	Value (Unit: mm)
*l_b_*	2.18
*b*	1.40
*t*	0.31
*l*	3.59
*w*	0.56

**Table 2:** Resonant frequencies of the first 12 eigenmodes of QTF.

Mode No.	Frequency/kHz	Mode No.	Frequency/kHz
1	8.939	7	117.55
2	16.443	8	137.50
3	26.796	9	137.81
4	31.380	10	162.53
5	51.122	11	183.39
6	82.308	12	215.76

**Table 3:** Comparison between calculation and harmonic simulation results of resonant characteristics.

Parameters	Calculation	Harmonic Simulation
*f*_1_/kHz	31.38	31.38
*f*_2_/kHz	183.39	183.40
*Q*_1_	7839	7550
*Q*_2_	11,520	12,694
*A*_1_/*A*_2_	11.95	11.41

**Table 4:** Resonant characteristics of QTF obtained by calculations, simulation, and experimental tests.

Parameters	Calculation	Simulation	Test 1	Test 2	Test 3
*f*_1_/kHz	31.38	31.38	32.758	32.759	29.755
*f*_2_/kHz	183.39	183.40	189.463	189.404	169.485
*Q*_1_	7839	7550	8190	8120	5110
*Q*_2_	11,520	12,694	11,710	17,376	8527
*A*_1_/*A*_2_	11.95	11.41	11.79	12.04	18.47

Test 1: QTF excited mechanically. Test 2: QTF excited electrically. Test 3: QTF with tips excited mechanically.

## Data Availability

The data presented in this study are available on request from the corresponding author.

## References

[1] Binnig G., Quate C.F., Gerber C. (1986). Atomic Force Microscope. Phys. Rev. Lett..

[2] Abrahamians J.O., Van L.P., Regnier S. (2016). Contributed Review: Quartz force sensing probes for micro-applications. Rev. Sci. Instrum..

[3] Hida H., Shikida M., Fukuzawa K., Murakami S., Sato K., Asaumi K., Iriye Y., Sato K. (2008). Fabrication of a quartz tuning-fork probe with a sharp tip for AFM systems. Sens. Actuators A-Phys..

[4] Chen Y.L., Xu Y.H., Shimizu Y., Matsukuma H., Gao W. (2018). High quality-factor quartz tuning fork glass probe used in tapping mode atomic force microscopy for surface profile measurement. Meas. Sci. Technol..

[5] Zhang Y.X., Li Y.Z., Song Z.H., Lin R., Chen Y.F., Qian J.Q. (2018). A High-Q AFM Sensor Using a Balanced Trolling Quartz Tuning Fork in the Liquid. Sensors.

[6] Kosterev A.A., Bakhirkin Y.A., Curl R.F., Tittel F.K. (2002). Quartz-enhanced photoacoustic spectroscopy. Opt. Lett..

[7] Hu Y.Q., Qiao S.D., He Y., Lang Z.T., Ma Y.F. (2021). Quartz-enhanced photoacoustic-photothermal spectroscopy for trace gas sensing. Opt. Express.

[8] Martínez N.F., Lozano J.R., Herruzo E.T., Garcia F., Richter C., Sulzbach T., Garcia R. (2008). Bimodal atomic force microscopy imaging of isolated antibodies in air and liquids. Nanotechnology.

[9] Jesse S., Kalinin S V., Proksch R., Baddorf A.P., Rodriguez B.J. (2007). The band excitation method in scanning probe microscopy for rapid mapping of energy dissipation on the nanoscale. Nanotechnology.

[10] Platz D., TholénE A., Pesen D., Haviland D.B. (2008). Intermodulation atomic force microscopy. Appl. Phys. Lett..

[11] Tetard L., Passian A., Thundat T. (2010). New modes for subsurface atomic force microscopy through nanomechanical coupling. Nat. Nanotechnol..

[12] Rodriguez B J., Callahan C., Kalinin S.V., Proksch R. (2007). Dual-frequency resonance-tracking atomic force microscopy. Nanotechnology.

[13] Garcia R., Herruzo E.T. (2012). The emergence of multifrequency force microscopy. Nat. Nanotechnol..

[14] Giessibl F.J. (1998). High-speed force sensor for force microscopy and profilometry utilizing a quartz tuning fork. Appl. Phys. Lett..

[15] Tung R.C., Wutscher T., Martinez-Martin D., Reifenberger R.G., Giessibl F., Raman A. (2010). Higher-order eigenmodes of qPlus sensors for high resolution dynamic atomic force microscopy. J. Appl. Phys..

[16] Oria R., Otero J., Gonzalez L., Botaya L., Carmona M., Puig-Vidal M. (2013). Finite Element Analysis of Electrically Excited Quartz Tuning Fork Devices. Sensors.

[17] Gonzalez L., Oria R., Botaya L., Puig-Vidal M., Otero J. (2015). Determination of the static spring constant of electrically-driven quartz tuning forks with two freely oscillating prongs. Nanotechnology.

[18] Gao F.L., Li X.D. (2015). Research on the Sensing Performance of the Tuning Fork-Probe as a Micro Interaction Sensor. Sensors.

[19] Lee M., Kim B., An S., Jhe W. (2019). Dynamic Responses of Electrically Driven Quartz Tuning Fork and qPlus Sensor: A Comprehensive Electromechanical Model for Quartz Tuning Fork. Sensors.

[20] Kim B., Jahng J., Khan R.M., Park S., Potma E.O. (2017). Eigenmodes of a quartz tuning fork and their application to photoinduced force microscopy. Phys. Rev. B.

[21] Zhang X.F., Gao F.L., Li X.D. (2018). Sensing Performance Analysis on Quartz Tuning Fork-Probe at the High Order Vibration Mode for Multi-Frequency Scanning Probe Microscopy. Sensors.

[22] Chen K., Liu Z., Xie Y., Zhang C., Xu G., Song W., Xu K. (2021). Numerical analysis of vibration modes of a qPlus sensor with a long tip. Beilstein J. Nanotechnol..

[23] Dagdeviren O.E., Miyahara Y., Mascaro A., Enright T., Grutter P. (2019). Amplitude Dependence of Resonance Frequency and its Consequences for Scanning Probe Microscopy. Sensors.

[24] Castellanos-Gomez A., Agrait N., Rubio-Bollinger G. (2011). Force-gradient-induced mechanical dissipation of quartz tuning fork force sensors used in atomic force microscopy. Ultramicroscopy.

[25] Labardi M., Lucchesi M. (2015). Split quartz tuning fork sensors for enhanced sensitivity force detection. Meas. Sci. Technol..

[26] Naber A. (1999). The tuning fork as sensor for dynamic force distance control in scanning near-field optical microscopy. J. Microsc.-Oxf..

[27] Hua B.C., Qian J.Q., Wang X., Yao J.E. (2011). Mechanical model of tuning forks used in scanning probe microscopes. Acta Phys. Sin..

[28] Jahng J., Potma E.O., Lee E.S. (2018). Tip-Enhanced Thermal Expansion Force for Nanoscale Chemical Imaging and Spectroscopy in Photoinduced Force Microscopy. Anal. Chem..

